# Dynamic Mercedes-Benz Sign in the Right Atrium

**DOI:** 10.31662/jmaj.2020-0025

**Published:** 2020-10-02

**Authors:** Ryo Tanabe, Hiroki Matsuura, Yuki Otsuka, Akira Endo

**Affiliations:** 1 Department of Surgery, Watanabe Hospital, Okayama, Japan; 2 Center for Graduate Medical Education, Okayama University Hospital, Okayama, Japan; 3 Department of General Internal Medicine, Okayama City Hospital, Okayama, Japan

**Keywords:** Dynamic Mercedes Benz sign, Venous air embolism, Venipuncture

An 87-year-old woman with an acute history of fever and cough presented to our emergency department. Non-contrast chest computed tomography (CT) revealed the unexpected presence of air in the right atrium (Figure 1A). Air embolisms in the heart are observed as the Mercedes-Benz sign ^(1)^, which is commonly observed in gallstones. This sign is not usually detected via electrocardiography-synchronized CT because its motion artifact is due to the heartbeat. They are detected via unenhanced chest CT in no less than 5.5% of asymptomatic patients after securing a peripheral venous line ^(2)^. Patients with a patent foramen ovale are at a risk of end-organ ischemia due to venous air embolism. Clinicians should be aware of the possible complication of venipuncture and should carefully follow-up patients who present with a Mercedes-Benz sign regardless of additional symptoms, such as paralysis. Our patient did not exhibit additional symptoms. Air was not observed on the follow-up CT 2 days later (Figure 1B).

**Figure 1.**
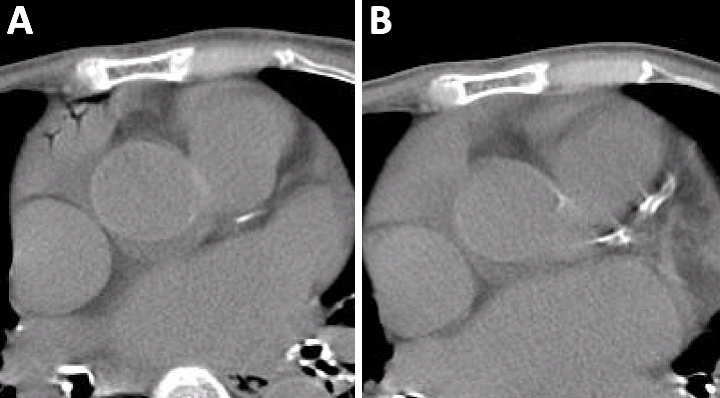
A. Electrocardiography-asynchronized computed tomography (CT) showing unexpected air (dynamic Mercedes-Benz sign) in the right atrium. B. Air was not observed on the follow-up CT performed 2 days later.

## Article Information

### Conflicts of Interest

None

### Author Contributions

Ryo Tanabe contributed to manuscript preparation, patient care, and discussion. Hiroki Matsuura, Yuki Otsuka, Akira Endo contributed to the discussion on the definitive diagnosis.

### Informed Consent

Informed consent has been obtained from the patient for the publication of their information, including photographs.

### Approval by Institutional Review Board (IRB)

This study did not require IRB approval.
